# Silencing UCHL3 enhances radio-sensitivity of non-small cell lung cancer cells by inhibiting DNA repair

**DOI:** 10.18632/aging.203043

**Published:** 2021-05-19

**Authors:** Miaowen Liu, Huimin Chen, Xinyue Chen, Jianping Xiong, Zhiwang Song

**Affiliations:** 1 Department of Oncology, The First Affiliated Hospital of Nanchang University, Nanchang, Jiangxi, People’s Republic of China; 2 Department of Hemodialysis, Nanchang First Hospital, Nanchang, Jiangxi, People’s Republic of China

**Keywords:** DNA damage, HR repair, NSCLC, radio-sensitivity, UCHL3

## Abstract

UCHL3 belongs to the UCH family and is involved in multiple biological processes. However, the biological functions and underlying mechanisms of action of UCHL3 in radio-sensitivity of non-small cell lung cancer (NSCLC) remain unknown. Here, we reported that the expression of UCHL3 was significantly up-regulated in NSCLC tissues and cell lines, and associated with poor prognosis of NSCLC patients. The expression of UCHL3 of NSCLC cells was increased after exposure to ionizing radiation (IR). Moreover, we found that knockdown of UCHL3 enhanced the radio-sensitivity of NSCLC cells both *in vitro* and *in vivo*. Furthermore, γH2AX foci staining and Western blot analysis showed that knockdown of UCHL3 increased IR-induced DNA damage. Knockdown of UCHL3 in NSCLC cells decreased homologous recombination (HR) repair efficiency and RAD51 foci formation. Collectively, our study revealed that knockdown of UCHL3 enhanced the radio-sensitivity of NSCLC cells and increased IR-induced DNA damage via impairing HR repair.

## INTRODUCTION

Lung cancer is a severe malignancy with high cancer-related mortality [1]. It has been widely accepted that approximately 85% of lung cancer cases are non-small cell lung cancer (NSCLC) [2, 3]. Radiotherapy is regarded as an effectual treatment for NSCLC [4]. Unfortunately, the great majority of patients with NSCLC have acquired. radio-resistance after receiving radiotherapy, which causes treatment failure [5]. Therefore, it is of utmost importance to elucidate the underlying molecular mechanism of radio-resistance and identify novel and effective approaches to improve radio-sensitivity.

Protein ubiquitination is involved in numerous biological processes, including cell cycle progression [6], inflammatory responses [7], DNA damage response (DDR) [8], signal transduction [9]. Moreover, regulation of ubiquitination events is closely related to deubiquitinases (DUBs), especially in DDR. For example, OTUB2 deficiency has been shown to cause RNF8 ubiquitination enhancement in DDR, thereby facilitating DNA double-stranded breaks (DSB) repair [10]. USP14 regulates DDR by affecting RNF168 ubiquitination [11], and USP7 influences RNF168 stabilization, thereby further regulating the recruitment of DDR downstream factors [12].

UCHL3 (EC 3.4.19.12), a deubiquitinase, has been related to mammalian oocyte maturation [13], human sperm count [14], and preimplantation embryo development [15], and antiviral response [16]. Moreover, UCHL3 participates in the initiation and progression of tumors. For instance, the progression of pancreatic cancer is promoted by UCHL3-FOXM1 axis [17]. In ovarian cancer, UCHL3 deubiquitinates TRAF2 to facilitate tumor progression [18]. Notably, UCHL3 overexpression renders tumor cell resistance to chemotherapy in breast cancer [8]. Nevertheless, the function and mechanism between UCHL3 and radio-sensitivity in NSCLC remain unknown.

Here, we reported that UCHL3 was up-regulated in NSCLC, and the increased expression of UCHL3 was significantly associated with poor prognosis of NSCLC patients. Furthermore, the expression of UCHL3 was increased after exposure to ionizing radiation (IR). Knockdown of UCHL3 enhanced the radio-sensitivity of NSCLC cells both *in vitro* and *in vivo*. Moreover, UCHL3 knockdown potentiated IR-induced DNA damage through hindering homologous recombination (HR) repair. Collectively, our results indicated that UCHL3 knockdown enhanced the radio-sensitivity of NSCLC cells by enhancing IR-induced DNA damage in an HR repair impediment manner.

## RESULTS

### UCHL3 was up-regulated in NSCLC and was associated with poor prognosis of NSCLC patients

**Figure 1:**
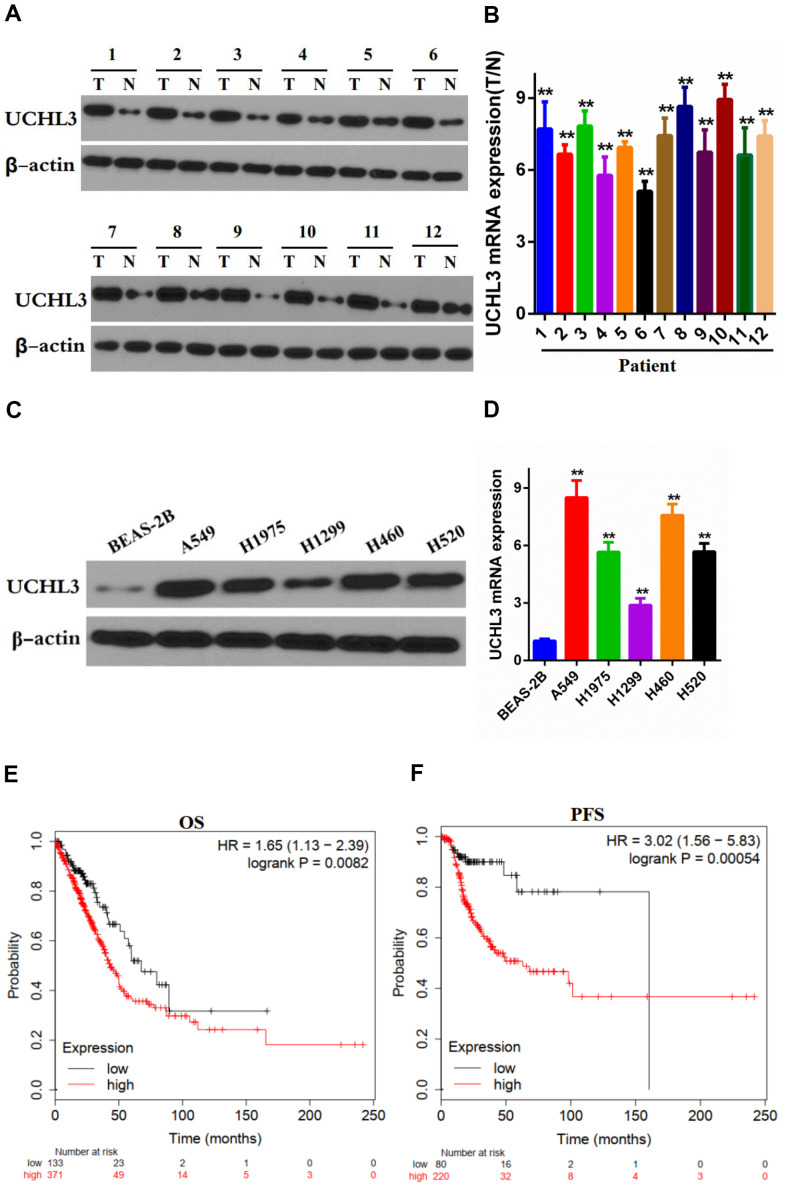
**Expression of UCHL3 is up-regulated in non-small cell lung cancer tissues and cell lines.** (**A**, **B**) Western blot analysis (**A**) and real-time PCR (**B**) analysis of UCHL3 expression in 12 paired tumor (T) tissues and adjacent non-tumor (N) tissues. (**C**, **D**) Western blot analysis (**C**) and real-time PCR (**D**) analysis of UCHL3 expression in normal lung epithelial cell lines BEAS-2B and non-small cell lung cancer (NSCLC) cell lines A649, H1975, H1299, H460, H520. (**E**, **F**) Kaplan-Meier analysis of OS (**E**), PFS (**F**) in NSCLC patients. Data was obtained from Kaplan-Meier Plotter website. OS, overall survival. PFS, progression-free survival. β-actin was used as a loading control. **P < 0.01.

Western blot analysis and RT-qPCR were performed to determine the expression of UCHL3 in non-cancerous tissues and NSCLC tissues. Our data showed that the expression of UCHL3 was notably up-regulated in NSCLC tissues when compared with non- cancerous tissues at both the protein and mRNA levels (Figure 1A, 1B). In addition, protein and mRNA expression levels of UCHL3 in NSCLC cell lines were significantly higher compared to that in normal lung epithelial cell BEAS-2B (Figure 1C, 1D). Moreover, assessment of the Kaplan-Meier plotter (http://kmplot.com/analysis/) demonstrated that UCHL3 overexpression was associated with poor overall survival (OS, HR=1.65, logrank P=0.0082) and progression-free survival (PFS, HR=3.02, logrank P=0.00054) (Figure 1E, 1F), which indicated that increased expression of UCHL3 was associated with poor prognosis of patients with NSCLC.

### Silencing of UCHL3 enhanced the radio-sensitivity of NSCLC cells

**Figure 2:**
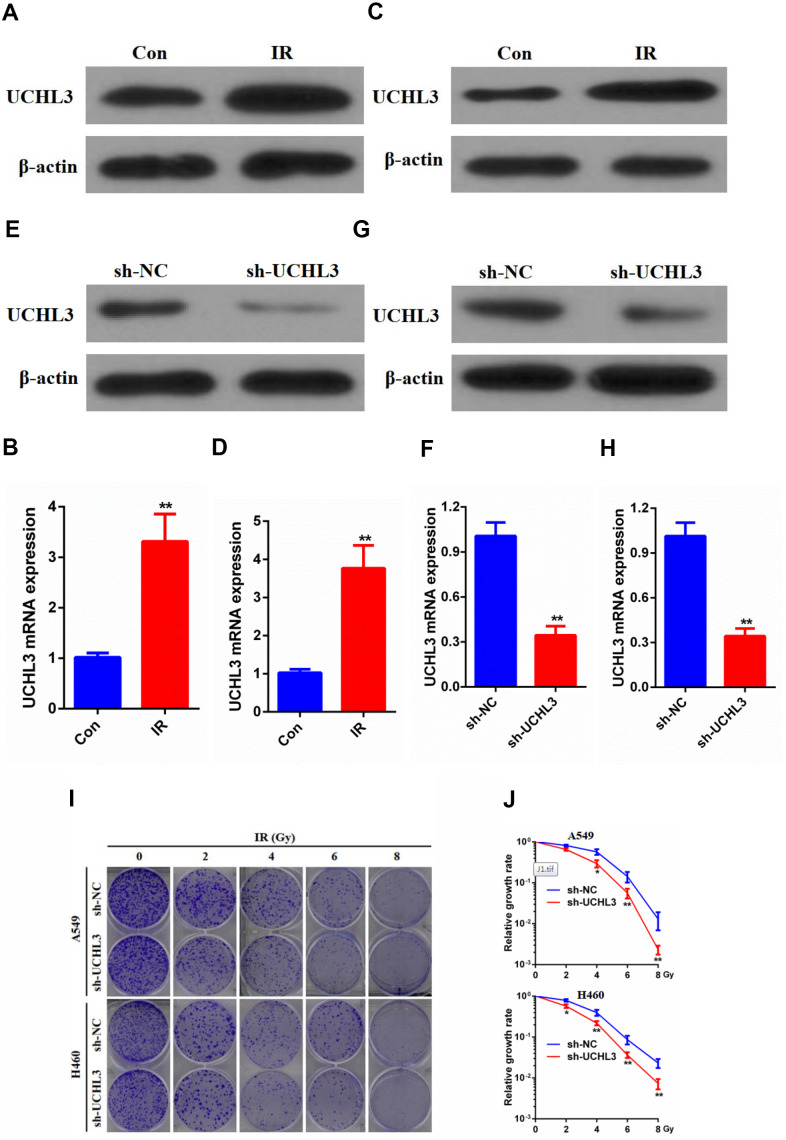
**UCHL3 knockdown enhanced radio-sensitivity of non-small cell lung cancer cells *in vitro*.** (**A**, **B**) Western blot analysis (**A**) and real-time PCR (**B**) analysis of UCHL3 expression in A549 control cells and cells exposed to ionizing radiation (IR). (**C**, **D**) Western blot analysis (**C**) and real-time PCR (**D**) analysis of UCHL3 expression in H460 control cells and cells exposed to IR. (**E**, **F**) Western blot analysis (**E**) and real-time PCR (**F**) analysis of UCHL3 expression in A549 cells transfected with sh-NC or sh-UCHL3. (**G**, **H**) Western blot analysis (**G**) and real-time PCR (**H**) analysis of UCHL3 expression in H460 cells transfected with sh-NC or sh-UCHL3. (**I**) Colony formation assay of A549 cells and H460 cells were transfected with sh-NC or sh-UCHL3 and received the corresponding radiation does (0, 2, 4, 6, 8 Gy). (**J**) Relative growth rate in A549 cells and H460 cells has shown. The relative growth rates are reported as the mean ± s.d. from three independent experiments. β-actin was used as a loading control. **P < 0.01.

UCHL3 was significantly up-regulated after exposure to IR as shown by Western blot analysis and RT-qPCR in A549 cells (Figure 2A, 2B) and H460 cells (Figure 2C, 2D). The Western blot and RT-qPCR results indicated that the expression of UCHL3 was remarkably decreased after transfection with shRNA-UCHL3 in A549 cells (Figure 2E, 2F) and H460 cells (Figure 2G, 2H) when compared with negative controls. Furthermore, IR treatment inhibited colony formation. The ability of colony formation was further inhibited after silencing of UCHL3 in A549 cells and H460 cells (Figure 2I, 2J). Taken together, these results showed that knockdown of UCHL3 enhanced the radio-sensitivity of NSCLC *in vitro*.

### Combining IR with UCHL3 knockdown effectively inhibited the growth of NSCLC cells *in vivo*

**Figure 3:**
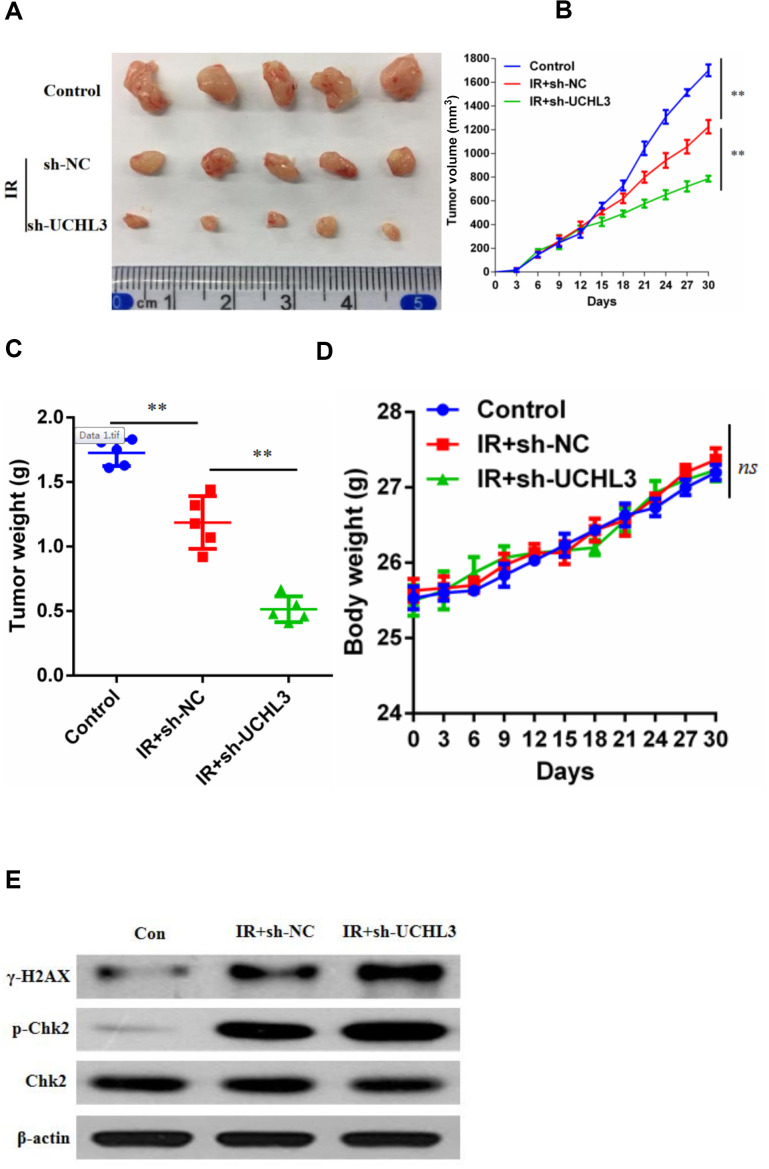
**UCHL3 knockdown enhanced radio-sensitivity of non-small cell lung cancer cells *in vivo*.** (**A**) Images of dissected tumors from nude mice. (**B**, **C**) Tumor growth curve and (**B**) tumor weight (**C**) are shown. (**D**) Body weight of mice treated with vehicle control, combination of transfected with sh-NC and radiation, combination of transfected with sh-UCHL3 and radiation. (**E**) Western blot analysis of γ-H2AX, p-Chk2, Chk2 after H460 cells treated with indicated treatment. β-actin was used as a loading control. Data are presented as the mean ± s.d. of five mice per group. **P < 0.01, n.s. no significance.

Since UCHL3 knockdown intensified the sensitivity of NSCLC cells to IR *in vitro*, xenograft models were established to assess the influence of a combination of IR and silencing UCHL3 *in vivo*. As shown in Figure 3A–3C, IR treatment alone inhibited tumor growth. Moreover, the combination of UCHL3 silencing and IR significantly decreased both tumor volume and tumor weight when compared to IR exposure alone. Furthermore, mouse body weight was not significantly different between the three groups, which implied that combination of IR and UCHL3 knockdown did not induce significant toxicity (Figure 3D). The expression of γH2AX and phosphorylated-ChK2 was up-regulated after exposure to IR, and silencing of UCHL3 further enhanced levels of γH2AX and phosphorylated-ChK2 (Figure 3E). Taken together, these results demonstrated that UCHL3 knockdown enhanced IR-induced DNA damage *in vivo*.

### Silencing of UCHL3 potentiated IR-induced DNA damage

**Figure 4:**
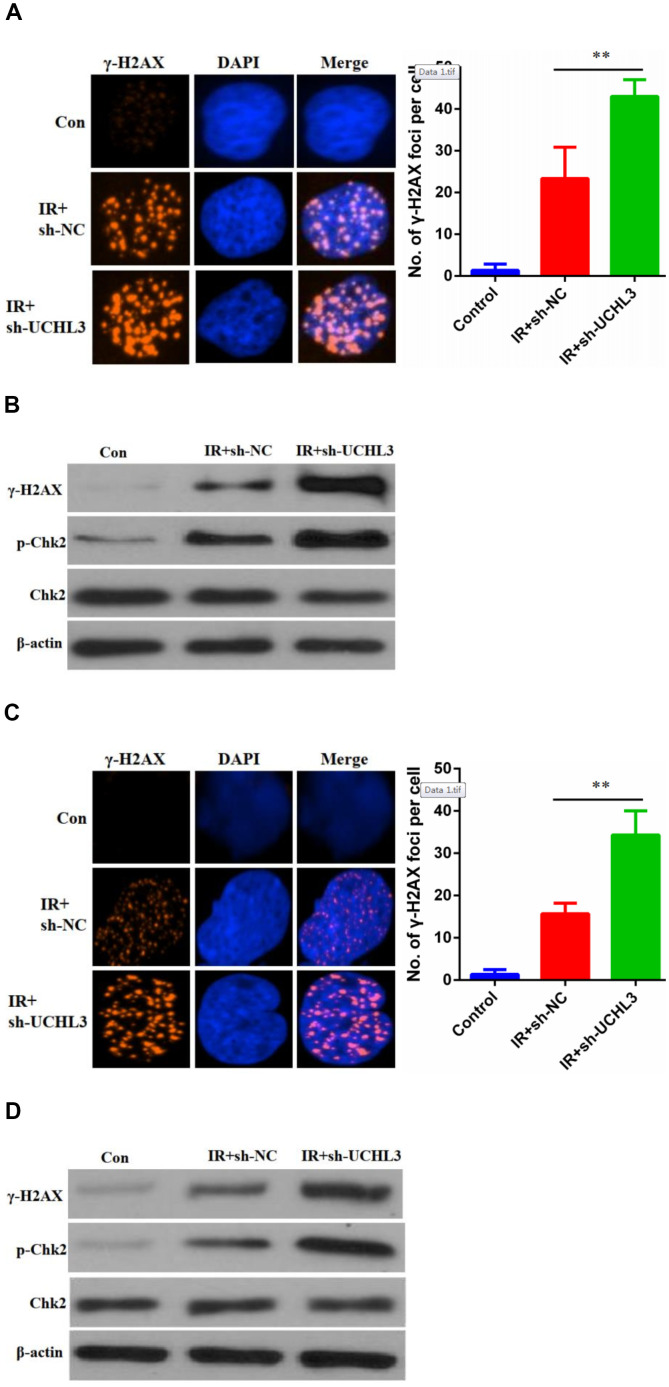
**UCHL3 knockdown enhanced ionizing radiation-induced DNA damage.** (**A**) γ-H2AX staining in A549 cells transfected with sh-NC and sh-UCHL3 after exposure to ionizing radiation (IR). (**B**) Western blot analysis of γ-H2AX, p-Chk2, Chk2 after A549 cells treated with indicated treatment. (**C**) γ-H2AX staining in H460 cells transfected with sh-NC and sh-UCHL3 after exposure to IR. (**D**) Western blot analysis of γ-H2AX, p-Chk2, Chk2 after H460 cells treated with indicated treatment. β-actin was used as a loading control. **P < 0.01.

Next, we investigated whether enhanced radio-sensitivity of UCHL3 knockdown was related to DNA damage. γH2AX, phosphorylated H2AX, was considered a hallmark of DNA-DSBs and was used to assess levels of DNA damage. The number of γH2AX foci in A549 cells treated with a combination of silencing of UCHL3 and IR was significantly increased when compared to IR exposure alone (Figure 4A). The expression levels of γH2AX and phosphorylated-ChK2 were up-regulated after exposure to IR in A549 cells, and silencing of UCHL3 in A549 cells further enhanced the levels of γH2AX and phosphorylated-ChK2 (Figure 4B). Similar results were observed in H460 cells (Figure 4C, 4D). Thus, these results demonstrated that UCHL3 knockdown enhanced IR-induced DNA damage.

### Silencing of UCHL3 impaired HR repair

**Figure 5:**
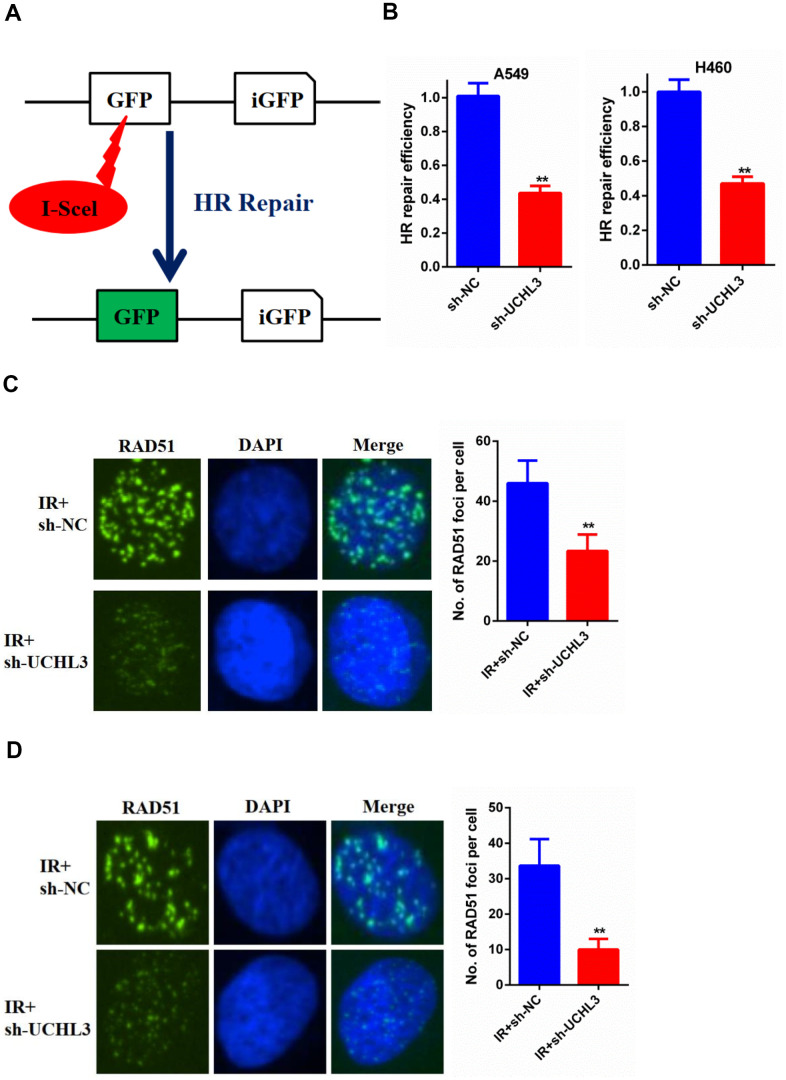
UCHL3 knockdown impaired HR repair (**A**) Schematic diagram of HR reporter system. (**B**) Analysis of HR repair activity in A549 cells and H460 cells after transfected with sh-NC or sh-UCHL3. (**C**) Analysis of RAD51 foci in A549 cells transfected with sh-NC and sh-UCHL3 after exposure to radiation. (**D**) Analysis of RAD51 foci in H460 cells transfected with sh-NC and sh-UCHL3 after exposure to radiation. **P < 0.01.

DSB was the major reason of IR-induced cell death, while HR was one of the main repair pathways for DSB. Therefore, we used an HR report assay to assess the HR repair efficiency (Figure 5A). Our data showed that knockdown of UCHL3 in NSCLC cells significantly decreased the HR repair efficiency (Figure 5B). Next, we explored the underlying mechanism between UCHL3 and HR repair. Our data showed that the number of RAD51 foci was significantly decreased in A549 cells in which UCHL3 was knocked down when compared with control cells (Figure 5C). In H460 cells, decreased RAD51 foci formation was observed in the sh-UCHL3 group (Figure 5D). Together, these data demonstrated that UCHL3 knockdown impaired HR repair by inhibiting the recruitment of RAD51 to DSBs.

## DISCUSSION

UCHL3 is a subset of deubiquitinating enzymes, belonging to the ubiquitin c-terminal hydrolase family [19]. In recent years, the relationship between UCHL3 and DNA damage repair has received increased attention. UCHL3 modulates DNA break repair via regulating TDP1 proteostasis [20]. Depletion of UCHL3 leads to a reduced number of KU80 foci after IR treatment, thereby impairing DNA repair [21]. UCHL3 deubiquitinates RAD51 and potentiates the connection between BRCA2 and RAD51, thereby facilitating DNA damage repair [8]. In addition, overexpression of UCHL3 causes tumor cell resistance to cancer treatment by increasing the DNA damage repair ability, whereas UCHL3 deficiency renders cells to be sensitive to this treatment [8]. Perifosine, a novel UCHL3 inhibitor, reinforces efficacy of PARP inhibitor in the treatment of triple-negative breast cancer via impairing homologous recombination [22]. Ouyang et al*.* reported that in lung cancer, UCHL3 is an AhR DUB that promotes lung cancer proliferation, tumor growth, and tumor stem-like properties through stabilizing AhR by its deubiquitination [23]. However, the biological function and underlying molecular mechanism of UCHL3 in the radio-sensitivity of NSCLC are still unknown.

Here, we found that UCHL3 was remarkably up-regulated in clinical NSCLC samples and cell lines. Patients who presented with an up-regulation of UCHL3 had a poorer prognosis, which was in accordance with the OS and PFS, as analyzed by Kaplan-Meier Plotter. Moreover, the expression of UCHL3 in NSCLC cells increased after exposure to IR. Additionally, knockdown of UCHL3 enhanced the radio-sensitivity of NSCLC cells both *in vitro* and *in vivo*.

Causing DNA damage, especially DNA DSBs, of tumor cells is the main mechanism of radiotherapy [24]. IR-induced DNA damage triggers the repair of cellular DNA damage [25]. Enhanced DNA repair abilities renders tumor cell resistance to radiotherapy [26]. CHAF1B decreases radio-sensitivity by enhancing DNA damage repair [27]. In turn, the inhibition of DNA damage repair enhances the radio-sensitivity of cancer cells [28]. OUT4 potentiates the radio-sensitivity of NSCLC via inhibiting DNA damage repair [29]. Thus, DNA damage repair is an important determinant of tumor development and outcome after radiotherapy [30]. Therefore, DNA damage repair efficiency has become an important factor limiting the efficacy of radiotherapy. HR is one of the key approaches for DNA damage repair [31, 32]. HR-based DNA repair is associated with radio-sensitivity [33]. hMOF reduction and TTK inhibition enhance radio-sensitivity of cancer cells through HR [34, 35]. Our results indicated that knockdown of UCHL3 promoted accumulation of IR-induced DNA damage and enhanced radio-sensitivity by impairing HR.

Finally, we explored the underlying mechanism between knockdown of UCHL3 and impairing HR. In a previous study, RAD51, which is acknowledged as a chain exchanging protein, is an important role in HR [36]. RAD51 affects the process of loading into single-stranded DNA, thereby leading to strand invasion in HR repair [37, 38]. It was reported that UCHL3 can promote RAD51-BRCA2 interaction and RAD51 foci formation through deubiquitinating RAD51 [8]. Thus, we further explored whether the increased radiosensitivity of NSCLC after UCHL3 knockdown was associated with inhibition of the formation of RAD51 foci. The results indicated that knockdown of UCHL3 decreased the formation of RAD51 foci. Therefore, knockdown of UCHL3 impaired the HR process by compromising the function of RAD51.

In summary, our findings demonstrated that UCHL3 was up-regulated in NSCLC, and that increased expression of UCHL3 was associated with poor prognosis of NSCLC patients. Moreover, UCHL3 knockdown enhanced the radio-sensitivity of NSCLC cells by increasing IR-induced DNA damage and impairing HR repair. Collectively, these results revealed that UCHL3 may be a novel promising therapeutic target against NSCLC.

## MATERIALS AND METHODS

### Patient tissue samples

In total, 12 paired NSCLC tissues and adjacent normal lung tissues were obtained from patients who received treatment at the First Affiliated Hospital of Nanchang University (Nanchang, China) and gave signed written informed consent. NSCLC diagnoses were confirmed by histopathological evaluation. All tissues were stored at -80° C. This study was approved by the Research Ethics Committee of the First Affiliated Hospital of Nanchang University (Nanchang, China).

### Cell lines and culture

Human lung cancer cell lines A549, H1975, H1299, H460, H520, and normal lung epithelial cell line BEAS-2B were purchased from ATCC. Human lung cancer cell lines and BEAS-2B cells were cultured in RPMI-1640 medium (Sigma, St. Louis, MO, USA) and BEGM medium (Lonza, Switzerland) respectively containing 10% FBS in an incubator at 5% CO_2_ and 37°.

### Western blot analysis and antibodies

Proteins were separated by SDS-PAGE and transferred to Hybond membranes (Amersham, Shanghai, China). Membranes were blocked by 5% nonfat milk and incubated with primary antibodies in TBST overnight at 4°. After washing three times in PBS, Hybond membranes were incubated with secondary antibodies conjugated to horse radish peroxidase for 1 hour at room temperature. Proteins were visualized by an enhanced chemiluminescence system (EMD-Millipore, Billerica, MA, USA). The antibodies used were as follows: anti-UCHL3 antibody (12384-1-AP), anti-γ H2AX antibody (A300-081A), anti-phospho-Chk2 antibody (Thr68), anti-Chk2 antibody (sc-17747), anti-β-actin antibody (ab8227).

### Quantitative reverse transcription PCR (RT-qPCR)

Total RNA was extracted from practical cell lines and tissues using TRIzol reagent (Invitrogen) according to the manufacturer’s guidelines. In brief, a total of 500ng RNA was reverse transcribed into cDNA for PrimeScript RT reagent Kit (TaKaRa). RT-qPCR was performed to determine UCHL3 levels using SYBR Premix Ex Taq (TaKaRa). β-actin served as the internal control, and the 2^-ΔΔt^ method was used to calculate relative expression.

### Transfection of shRNA

To knockdown UCHL3, transfection with shRNA was performed. The control sh-RNA sequence and the UCHL3 target sequence were synthesized and designed by Shanghai Gene Chemical Technology Co., Ltd., (Shanghai, China). The transfection approach was employed by a Lipofectamine 3000 kit (Thermo Fisher Scientific Inc.) following the manufacturer’s guidelines. A549 cells and H460 cells were seeded in 24-well plates, and lentiviral vector solution was used for culturing and incubating the cells.

### Colony formation assay

A549 cells and H460 cells were seeded into 6-well plates and cultured overnight. Subsequently, cells were divided in different groups and received radiation doses of 0, 2, 4, 6, and 8 Gy, respectively. Ten days later, cells were washed with PBS, fixed in methanol, stained with crystal violet, and the number of colonies was counted. Finally, the data were put in a linear quadratic model to create relative growth rate curves.

### *In vivo* study

Female nude mice (5-week old) were randomly divided into three groups. A total of 1×10^6^ A549 cells were injected in group 1. Next, 1×10^6^ A549 cells transfected with scramble RNA or UCHL3 shRNA were respectively injected in groups 2 and 3. All injections were given by subcutaneous administration. Three groups of mice grew up in the same environment. Mice in group 1 did not receive any treatment, and served as a control. Mice in groups 2 and 3 received radiation after 6 days. Tumor sizes and mouse weight were assessed and recorded every three days. Tumor volumes were calculated by the following formula: 0.5× (length × width^2^).

### Immunofluorescence staining

Three groups of cells (control, shRNA-NC, shRNA-UCHL3) were respectively seeded on coverslips and underwent IR. One day later, cells were washed with PBS, fixed in 3% paraformaldehyde, and treated with 0.5% Triton-X. Cells were blocked in 5% goat serum and incubated with anti-γH2AX or RAD51 antibodies overnight. The next day, cells were washed with PBS and incubated with secondary antibodies for 1 hour, and cell nuclei were counterstained with DAPI. Cells were visualized by confocal microscopy.

### HR repair assay

Negative control or UCHL3 knockdown DR-GFP cell lines were constructed in both A549 cells and H460 cells. In brief, cells were transfected with the vector expressing I-SCEI. The condition of recombinant cells was determined by flow cytometry 2 days later.

### Statistical analysis

Data were presented as the mean ±SD from at least three independent experiments and analyzed with GraphPad Prism software (GraphPad Inc., USA). The statistical discrepancy between groups was evaluated by the t-test or χ2 test. *P* < 0.05 was considered statistically significant.
